# 

**Published:** 1858-06

**Authors:** 


### CONTENTS.

### ORIGINAL COMMUNICATIONS.

PAGE.

ARTICLE I.—Address—Introductory to the Fourth Course of Lectures in the Atlanta Medical College. By W. F. WESTMORELAND, M. D., Professor of the Principles and Practice of Surgery,........................................579

ARTICLE II.—Abuse of the Pessary. By J. S. WEATHERLY, M. D., Montgomery, Ala........................................590

ARTICLE III.—Complicated case of Obstetrics. By B. T. SMITH, M. D,, Central Institute, Ala........................................593

### SELECTIONS.

Observations upon Otorrhea as a Sequela to Scarlet Fever,....................596

Removal of Urinary Calculi from Right Lumbar and Pubic Regions, by incision, 606

St. Louis Medical Society,....................609

Report to the State Society of Michigan on the Diseases of Children,..............614

On the Constitutional Effects prodnced by Substances introduced into the Urethra,....................622

### EDITORIAL AND MISCELLANEOUS.

American Medical Association,....................657

Atlanta Medical College,....................658

Bibliographical,....................659

American Medical Association—Eleventh Annual Meeting,....................660

Receipts,....................672

